# The influence of environmental cognition on green consumption behavior

**DOI:** 10.3389/fpsyg.2022.988585

**Published:** 2022-09-27

**Authors:** Chi Xie, Ru Wang, Xiaoxiao Gong

**Affiliations:** ^1^School of Business, Hunan Normal University, Changsha, China; ^2^Research Institute of Economics and Management, Southwestern University of Finance and Economics, Chengdu, China; ^3^School of Management, Guizhou University, Guiyang, China

**Keywords:** green consumption, theory of planned behavior, environmental cognition, attitudes toward green consumption, green consumption subjective norms, green consumption perceived behavioral control

## Abstract

With rising consumption and environmental problems, there is an increasing need for green consumption. From a micro perspective, the influence of environmental cognition on consumers’ green consumption behaviors and the related mechanisms are examined through multilayer linear analysis and 2010 China General Social Survey (CGSS) microdata with the theory of planned behavior (TPB) as the model framework. The study shows that (1) environmental cognition positively influences attitudes toward green consumption, green consumption subjective norms, and green consumption perceived behavioral control, which leads to increased intentions to engage in green consumption and actual green consumption behaviors. Environmental cognition can either promote the intention toward and lead to green consumption behavior or directly promote green consumption behavior. (2) The more developed a region’s economy is, the stronger people’s attitudes toward green consumption will be; additionally, the greater the perceived external pressure to engage in green consumption becomes, the greater the likelihood that people will develop the intention to engage in green consumption behavior. Regional environmental quality inhibits green consumption intention to a certain extent. (3) The influence of environmental cognition on green consumption shows regional heterogeneity.

## Introduction

The rise in consumption and the growing prominence of environmental issues are two important changes in the current development of Chinese society. On the one hand, consumption has become the leading driver of China’s economic growth in recent years, with final consumption expenditures contributing 65.4% to gross domestic product (GDP) growth in 2021 (Xinhua, 2022). On the other hand, while people are enjoying an increasingly affluent life, they are also facing serious problems, such as environmental pollution and increasing carbon emissions, which are also problems worldwide. As people demand a higher-quality ecological environment and their own health improves, consumption plays a more important role in environmental protection.

The overall level of green consumption in society is shaped by many individual daily consumption-specific behaviors with highly individual characteristics, and many studies have been conducted on green consumption behavior from a micro perspective (Choi and Johnson, 2019; Do Paço et al., 2019). It has been argued that individual green consumption behavior is mainly influenced by two factors—consumer subjects (consumers) and consumer objects (products and services). Consumer subject factors include consumers’ environmental attitudes (Al Mamun et al., 2018); perceptions of social, political, and legal changes (Leonidou et al., 2010); and the ability and willingness to pay (Steg and Vlek, 2009). Consumer object factors include product eco-labeling (Chen, 2010), green product quality (Joshi and Rahman, 2015), and green product availability (Vermeir and Verbeke, 2006). However, most of the above factors are governed by objective levels of economic and technological development, except for consumers’ environmental attitudes, which are often influenced by their own environmental cognition. Environmental cognition refers to how individuals structure their thinking about environmental issues and associated political actions (Henry and Dietz, 2012). When viewed from a consumption perspective, environmental cognition represents consumers’ understanding of the environment and its underlying relationships with significant ecological implications that lead to changes in environmental perspectives. However, due to the existence of attitude-behavior inconsistencies in green consumption behavior, among other factors, the mechanisms by which consumers ultimately make decisions about green consumption are complex, especially when environmental cognition is considered, and the mechanisms of how exactly environmental cognition affects green consumption behavior are unclear and must be further refined.

In this paper, we analyze the relationship between environmental cognition and green consumption behavior based on the theory of planned behavior (TPB), which assumes that individuals are rational and that individuals’ consumption behavior intentions are influenced by their consumption attitudes, subjective norms of consumption, and perceived behavioral control of consumption (Ajzen, 1985). Moreover, the TPB has been widely applied to explain and predict individual behavior. In green consumption research, the theoretical model of planned behavior is often extended to some extent for different research subjects and is found to better explain the mechanisms by which consumer green consumption behavior arises (Teng et al., 2013; Maichum et al., 2016; Yadav and Pathak, 2017). Therefore, this paper analyzes and tests how environmental cognition influences attitudes toward green consumption, green consumption subjective norms, and green consumption perceived behavioral control through a TPB model and further promotes consumers’ intentions and behaviors regarding green consumption.

The main contributions of this paper are as follows. First, by expanding the theoretical model of planned behavior, the mechanism of the influence of environmental cognition on green consumption behavior is clarified, and it is argued that environmental cognition positively influences attitudes toward green consumption, green consumption subjective norms, and green consumption perception behavioral control. This influence leads to intentions to engage in green consumption and green consumption behavior and ultimately to green consumption behavior by either promoting intentions to engage in green consumption or directly promoting green consumption behavior. Second, based on a microsample from China, combined with a multilayer linear analysis, the model portrays the differentiated causal relationships among different regional samples, making the empirical results more reliable and nuanced. In this paper, instrumental variables (IVs) of the geographic environment are selected to minimize the effects of endogeneity issues and to ensure unbiased parameter identification.

## Literature review and hypothesis development

### Environmental cognition

Many studies regard cognition as the important basis of behavior and think that behavior is often based on people’s cognitive level. Some studies have argued that environmental cognition is an important reason for environmental protection. Studies have found that environmental cognition is the most favorable predictor of environmentally friendly behavior (Hines et al., 1987; Gifford and Nilsson, 2014). However, other studies have found that environmental cognition has little relationship with environmental protection behavior. Frick et al. (2004) argued that there is no correlation between environmental cognition and ecological behavior; alternatively, the relationship is weak (Gamba and Oskamp, 1994; Grob, 1995). In addition, environmental cognition and behavior are inconsistent from time to time. Some scholars have pointed out that this inconsistency may be because environmental cognition involves multiple dimensions and its contents should be distinguished (Ellen, 1994). Individual consumption decisions are unilaterally explained based on cognitive factors alone, and theoretical support or identification of attitudes, external pressure, and other key intermediary variables is necessary (Carmi et al., 2015; Ertz et al., 2016). Since the influence of environmental cognition on environmental protection behavior is very complex, to better promote environmental protection behavior, scholars are very concerned about how environmental cognition can be better transformed into environmental protection behavior.

### Green consumption behavior

Scholars have given different definitions to green consumption. From the perspective of commodities, green consumption refers to purchasing environmentally friendly products to avoid damaging the environment (Chan, 2001). From the perspective of consumers, it refers to the behavior of an individual who considers environmental or social issues while making purchasing or nonpurchasing decisions (Gilg et al., 2005; Peattie, 2010). From the perspective of the consumption life cycle, green consumption involves using green products as much as possible, and these products account for a small amount of the environment in the whole life cycle (including the production and post-use stages; Sheth et al., 2011). It can be seen that green consumption requires that commodities, consumers, and the use process of commodities should consider environmental factors. In the past, some scholars thought that green consumption was just a collection of green purchasing behaviors in different specific forms (Gilg et al., 2005). Subsequently, the academic community’s understanding of green consumption gradually increased to include the whole process of production, transportation, sales, use, and post-use. Akenji (2014) argued that green consumption refers to the production, publicity, and consumption of goods and services based on environmental protection. Tan et al. (2016) argued that green consumption behavior (GCB) includes recycling, protecting waterways, reducing shopping packaging, and purchasing and consuming environmentally friendly products. Scholars have also proposed other concepts similar to green consumption behavior, including sustainable consumption (Vermeir and Verbeke, 2006), green purchasing (Liu et al., 2012), environmentally friendly consumption (Strizhakova and Coulter, 2013), and pro-environmental consumption behavior (Mainieri et al., 1997). Green consumption is not a result of statutory control. Rather, it arises from the values held by consumers (Wu and Chen, 2014). Among many values, ecological cognition is an important content of values.

### Public-sphere and private-sphere pro-environmental behavior

Existing studies have analyzed pro-environmental behavior based on both the public and private spheres. Private-sphere pro-environmental behavior refers to the purchase, use, and disposal of personal and household products that have an environmental impact, such as eco-friendly purchasing, public transportation, or household energy consumption (Stern, 2000). Private-sphere pro-environmental behavior is relevant to most consumers and has direct environmental consequences (Ertz et al., 2016). Pro-environmental behavior in the public sphere is receiving more attention due to the spillovers of environmental behaviors. Public-sphere pro-environmental behavior is defined as behavior that affects the environment directly through committed environmental activism, such as active involvement in environmental organizations and demonstrations, or indirectly by influencing public policies, such as petitioning for environmental issues (Stern, 2000). Ertz et al. (2016) included three items to measure public-sphere behavior and found that there is no indirect effect of perceived busyness and wealth on either public or private behavior. This finding is consistent with Lee et al. (2014), who revealed the fact that consumers enact environmental activist behavior when they feel helpless about a given situation and view public activism as the ultimate recourse for solving problems. Public-sphere behaviors can be classified into activist and nonactivist behaviors. Stern (2000) found considerable empirical support for nonactivist behaviors and pointed out that the effects may be large because public policies can change the behaviors of many people and organizations at once. Hence, we can conclude that existing literature have found some evidence of public-sphere behaviors, which also illustrates the importance of investigating public policies.

### Influence of environmental cognition on green consumption behavior based on TPB

Many scholars use TPB models in their studies to analyze the factors and main mechanisms that influence individual behavior. The TPB was proposed by Ajzen as a deepening and expansion of the theory of rational behavior (TRB), which assumes that individuals are rational and that their willingness to behave is influenced by their attitudes toward behavior and external subjective norms (Fishbein and Ajzen, 1975). The TPB assumes that individual behavior is influenced not only by individual attitudes and subjective norms but also by individual perceptions regarding the ease of achieving certain behaviors, which is referred to as perceived behavioral control. The increasing research on consumption has shown that the mechanisms that generate individual consumption behavior can be better explained by expanding the TPB model (Wu and Chen, 2014; Kao and Tu, 2015; Huang et al., 2017; Emekci, 2019; Ertz et al., 2021). This model provides a reliable and detailed framework for the study of various behaviors, and it is no longer simply thought that attitudes directly generate behaviors.

Many studies rely on TPB model to test the impact of green consumption behavior in different fields, including the behavior of purchasing energy-efficient appliances (Bhutto et al., 2020), sustainable municipal organic waste management (Kopaei et al., 2021), green hotels (Kwon and Ahn, 2020), and reusable containers (Ertz et al., 2017). Besides, environmental cognition can have multiple effects on consumers’ green consumption behavior. Related studies have shown that personal environmental awareness and environmental knowledge have a positive impact on green consumption behavior (Sun et al., 2019). Corporate environmental awareness can significantly moderate the relationship between environmental issue disclosure and consumers’ willingness to participate in green consumption activities (Rustam et al., 2020). Although environmental knowledge and environmental awareness related to environmental cognition can influence consumers’ green consumption behavior through various pathways, the connotation of environmental cognition is much greater than environmental awareness and environmental knowledge. Environmental cognition refers to people’s awareness of environmental issues, their support for solving environmental problems, and their willingness to work hard. Environmental knowledge is only a factor in environmental cognition (Sun et al., 2019). Studies have not included environmental cognition in systematic theoretical models to analyze the mechanism of its influence on green consumption.

This paper analyzes the influence mechanism of environmental cognition on green consumption behavior based on the TPB framework. Consumption attitudes, subjective norms, and perceived behavioral control are three important variables in the TPB model. With the enhancement of environmental cognition and people’s demand for a better living environment, people possess more environmental cognition, which leads to their positive attitudes toward green consumption (Young et al., 2010). Subjective norms refer to individuals’ perceptions of pressure from reference groups. With the increasingly serious ecological and environmental problems driving people’s environmental awareness, calls from the public for a green consumption model are increasing daily. Increased external pressure pushes consumers to adopt green consumption, and subjective norms for consumers thus also increase. Perceived behavioral control refers to an individual’s perception of his or her own ability, which is related to the resources and abilities that he or she possesses and the degree of effort that he or she is willing to dedicate to a particular behavior. Improved environmental cognition causes consumers to care more about the environment. The desire for a good environment motivates consumers to engage in more green consumption behavior to protect the environment and enhances their perceived behavioral control. Therefore, this paper proposes the following hypotheses:

*H1*: Environmental cognition positively influences attitudes toward green consumption.

*H2*: Environmental cognition positively influences green consumption subjective norms.

*H4*: Environmental cognition positively influences green consumption perceived behavioral control.

Behavioral intention is considered a direct antecedent of behavior (Ajzen, 2002), and in most studied cases, TPB models support the idea that when consumers have green consumption intentions, they choose products and services that protect the environment accordingly (Yadav and Pathak, 2017). However, it is also necessary to test whether environmental cognition influences green consumption behavior through pathways other than those mentioned above. Environmental cognition is a quantitative manifestation of consumers’ environmental knowledge and awareness, and as consumers’ environmental cognition level increases, they become more concerned about environmental issues, more willing to acquire skills related to green consumption, and better able to understand the importance of green consumption for environmental protection and consumption development. Moreover, environmental cognition is helpful for stimulating people’s intentions and specific behaviors toward green consumption. Thus, consumers with high levels of environmental cognition also exhibit strong intentions to engage in green consumption and are correspondingly more likely to exhibit green consumption behaviors. Therefore, this paper proposes the following hypotheses:

*H4*: Environmental cognition positively influences intentions to engage in green consumption behavior.

*H5*: Environmental cognition positively influences green consumption behavior.

**Figure 1:**
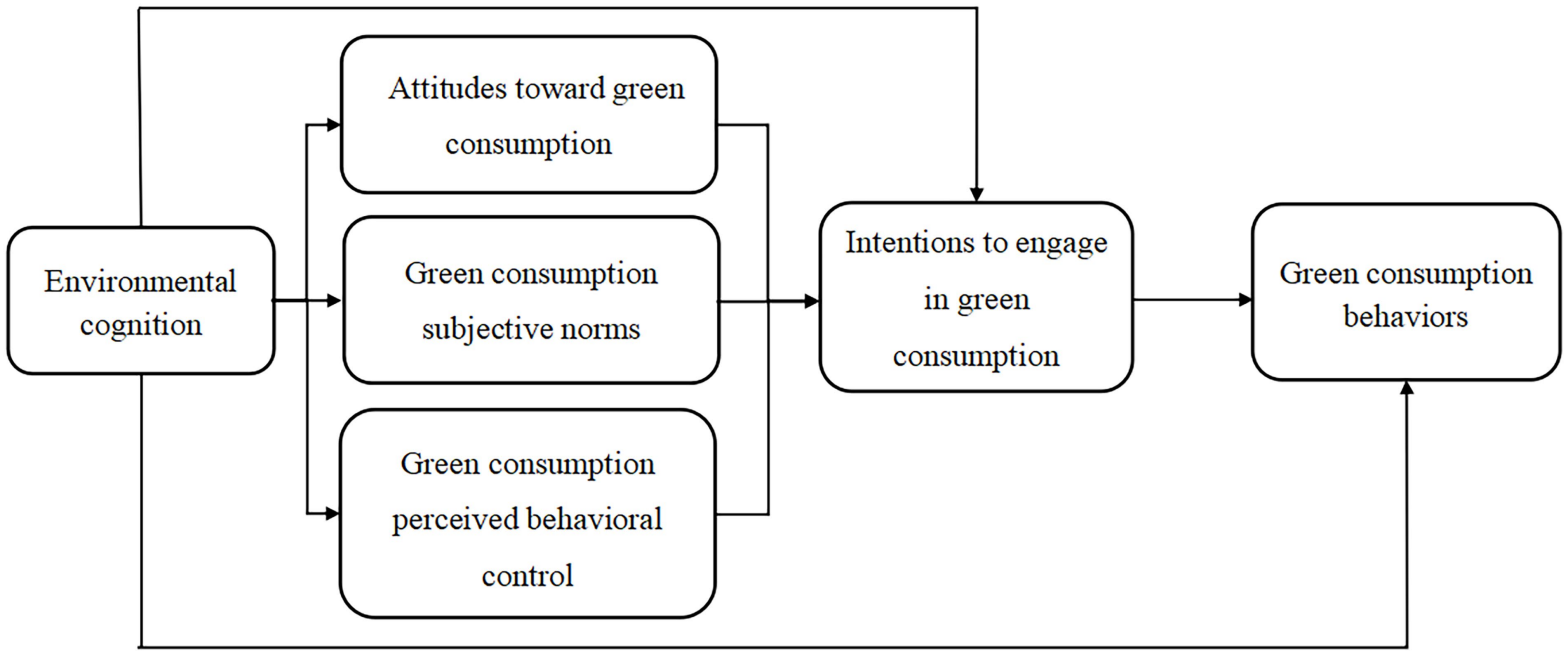
TPB model of green consumption behavior.

In summary, this paper aims to address the mechanism of green consumption behavior from the perspective of environmental cognition: Does environmental cognition have an impact on green consumption behavior? What are the pathways through which this influence is exerted? Based on the TPB, this work adds the antecedent variable of an individual’s “environmental cognition” and constructs an extended TPB of green consumption behavior (as shown in Figure 1). The clarification of this mechanism can help enrich the application of the TPB model in the field of green consumption and provides empirical evidence based upon which targeted countermeasures can be devised for the development of green consumption.

## Empirical test

### Data

The data used for this study are obtained from the 2010 China General Social Survey (CGSS) released by the China Survey and Data Center of Renmin University of China. The CGSS2010 has a more comprehensive environmental survey module (Part L), which is optional. Among the 11,783 valid samples, those born in February, September, November, and December completed the environmental questionnaire. After excluding missing values, the sample analyzed in this study included 3,240 respondents from 31 provincial administrative units. In the descriptive statistics and regression sections, CGSS sample weights are considered to ensure sample representativeness.

### Variables

There are two categories of factors that affect individual green consumption behavior: individual-level factors and provincial-level factors. Individual-level variables include an individual’s intentions to engage in green consumption behavior, green consumption behaviors, attitudes toward green consumption, green consumption subjective norms, green consumption perceived behavioral control, environmental cognition, and other control variables. Provincial-level variables include the regional economic level and the environmental level.

With regard to intentions to engage in green consumption behavior, the CGSS2010 included five questions to determine whether consumers have the intention to engage in green consumption and the strength of that intention. The final scale was obtained as shown in Table 1.

**Table 1:** Items related to green consumption intentions.

Original question number	Positive/negative	Question item	Assignment
1	Positive	Do you often refrain from buying certain products specifically for the sake of environmental protection?	Always, often, sometimes, and never are assigned values of 4, 3, 2, and 1, respectively.
2	Positive	Do you often make a point of buying fruits and vegetables that have not been treated with chemical fertilizers and pesticides?	
4	Positive	Do you often reduce the amount of energy or fuel you consume at home, including oil, gas, and electricity, specifically to protect the environment?	
5	Positive	Do you often conserve or reuse water specifically for the sake of the environment?	
6	Positive	Do you often purposely sort glass, aluminum cans, plastic or newspaper to facilitate recycling?		

The academic scope of green consumption has been extended from green products to green processes such as production, transportation, distribution, use, and post-use (Akenji, 2014). Therefore, the measurement of intentions to engage in green consumption should include consumers’ intentions in all aspects of green commodity selection, use, and post-use as much as possible. The original questions 1 and 2 in Table 1 measure the intensity of individual intentions to engage in green consumption behavior in the commodity selection process, the original questions 4 and 5 measure intentions to engage in the green consumption commodity use process, and the original question 6 measures intentions to be involved in the green consumption post-use process. The response options for each variable are “always,” “often,” “sometimes,” and “never,” the values of which are set to 4-1, respectively. The coefficient of reliability (Cronbach’s alpha) is 0.79 for the five questions, which shows a high degree of internal consistency. Furthermore, the five variables are summed and averaged to obtain green consumption behavior intention, and the higher the value, the stronger the individual’s green consumption behavior intention.

#### Green consumption behavior

The research has often shown that behavioral intentions lead to behaviors, and the CGSS 2010 question “Have you and your family taken any action to address the environmental problems that you and your family are experiencing?” can be used to directly describe whether green consumption behavior has occurred. Based on the context of the comprehensive environmental survey module (Part L) of the CGSS2010, behavior here mainly refers to consumption behavior. Due to technical-level constraints, consumption choice was the main way for families to participate in environmental protection in China around 2010. “Took action” is assigned a value of 4; “Did not take action” is assigned a value of 1; and “Tried to take action but did not know what to do” indicates a person’s willingness to do so but his or her inability to find a means of implementation, and it is assigned a value of 3. The higher the value of green consumption behavior, the more thorough an individual’s green consumption behavior, and the lower the value, the less thorough an individual’s green consumption behavior.

#### Attitudes toward green consumption

A representative variable of environmental attitudes is environmental concern (Wang et al., 2019). It has been argued that environmental concern refers to the extent to which people are aware of and support solutions to problems involving the ecological environment or their willingness to contribute to solutions to such problems (Dunlap and Robert, 2002). It has been shown that the greater the public concern about environmental-related issues such as green consumption, the more it is conducive to the formation of good green awareness, and the more likely it is that green consumption behavior will occur in daily life. This paper regards environmental concern as a variable to measure attitudes toward green consumption in the model. The Chinese version of the environmental concern scale (CNEP), which has been tested to show good reliability, is used and is based on 10 attitude judgment items in the CGSS2010, which are as follows: (1) The current total population is approaching the limit of what the earth can sustain. (2) Human destruction of nature often leads to catastrophic consequences. (3) Humans are currently abusing and destroying the environment. (4) Animals and plants have the same right to live as humans. (5) Nature’s self-balancing ability is strong enough to cope with the impact of modern industrial society. (6) Although humans have special abilities, they are still governed by the laws of nature. (7) The so-called “environmental crisis” faced by humans is an overstatement. (8) The earth is like a spaceship with only a limited amount of space and resources. (9) The balance of nature is fragile and can be easily disturbed. (10) If everything continues as it is now, humankind will soon suffer a serious environmental disaster. The Cronbach’s alpha coefficient for the above 10 attitude judgment items is 0.91, which indicates good internal consistency. Similarly, the responses for each item are assigned as “totally disagree,” “somewhat disagree,” “indifferent/cannot choose,” “agree” and “agree completely,” which are assigned scores of 1-5, respectively. Items (5) and (7) are assigned reverse scores according to the question formulation. The higher the score, the more concerned the respondent is about the ecological environment and the more positive his or her attitudes are toward green consumption.

#### Green consumption subjective norms

The formation of subjective norms is determined by the level of trust individuals have in what others think should be done and by their own level of motivation to maintain consistency with others’ opinions. According to the meaning of subjective norms assigned by the TPB, the green consumption subjective norm is the degree of external social pressure that consumers feel in their own consumption behavior, which reflects the influence of significant others or groups on their green consumption behavior decisions. Moreover, the stronger the external pressure perceived by consumers, the higher their intention to engage in green consumption will be. The CGSS2010 includes the following three statements: “People are somewhat overly concerned about the environmental damage caused by human progress,” “We worry too much about future environmental conditions and not enough about current prices and employment,” and “In modern life, almost everything we do is harmful to the environment,” which measure the subjective criterion of green consumption. Cronbach’s alpha coefficient for the three items is 0.72, which indicates good internal consistency. For the first two questions, the responses “completely disagree,” “agree less,” “do not agree or disagree,” “agree more,” and “totally agree” are assigned scores of 5-1, respectively. For the last statement, “Almost everything we do in modern life is harmful to the environment,” its answer items “totally disagree,” “somewhat disagree,” “do not agree or disagree,” “somewhat agree,” and “completely agree” are assigned scores of 1-5, respectively, and the answer “cannot choose” is assigned a score of 3. The green consumption subjective norm in the value domain of Al Mamun et al. (2018) and Sun et al. (2019) is obtained by summing the scores of the three question items. The higher the score is, the greater the influence and pressure of external norms on green consumption behavior.

#### Green consumption perceived behavioral control

Perceived behavioral control refers to the degree of difficulty individuals experience when performing a specific behavior and includes perceived control and perceived difficulty, both of which act independently on individual behavioral intentions and can also directly determine individual behavior. Consumers usually evaluate the feasibility and ease of green purchasing behavior when making decisions, and when they perceive that it is difficult to adopt green purchasing behavior, their purchase intention is weakened. Conversely, when they perceive that it is easy to adopt green purchasing behavior, their purchase intention is enhanced. According to the meaning of perceived behavioral control of the TPB, perceived behavioral control of green consumption refers to consumers’ perceived ease of performing green consumption behavior. Some studies have used the convenience of purchasing green food and whether purchasing green products is entirely the consumer’s choice as important variables in measuring perceived behavioral control factors (Qi and Ploeger, 2019). In general, individuals are more likely to engage in green consumption behavior when they perceive that their behavior is under their own control in terms of their willingness and ability. According to the CGSS2010, the following items can be used to measure green consumption perceived behavioral control: “It is difficult for people like me to do anything for environmental protection,” “It is difficult for me to figure out whether my current lifestyle is harmful or beneficial to the environment,” “There are more important things in life than protecting the environment,” “My efforts to protect the environment are meaningless unless everyone does the same,” “Many of the claims about environmental threats are exaggerated,” and “I will do what is good for the environment even if it costs more money and time.” A reliability test is conducted, and the Cronbach’s alpha coefficient for the six items is 0.75, which indicates good internal consistency. For the first five items, the responses “totally disagree,” “somewhat disagree,” “do not agree or disagree,” “somewhat agree,” and “totally agree” are assigned scores of 5-1, respectively, and the response “cannot choose” is assigned a score of 3. For the statement “Even if it costs me more money and time, I still want to do what is good for the environment,” the answer items “completely disagree,” “somewhat disagree,” “do not agree or disagree,” “somewhat agree,” and “completely agree” are assigned scores of 1–5, respectively, and the response “cannot choose” is assigned a score of 3. The scores of the six questions are summed to obtain green consumption perceived behavioral control in the value range of Chen (2010). The higher the score, the less difficult it will be for a consumer to engage in green consumption.

#### Environmental cognition

There are 10 basic statements in the CGSS2010 on ecological cognition: (1) “Automobile exhaust does not pose a threat to human health;” (2) “The excessive use of fertilizers and pesticides will damage the environment;” (3) “The use of phosphorus-containing laundry detergent will not cause water pollution;” (4) “Fluorine emissions from fluoride-containing refrigerators will destroy the ozone layer of the atmosphere;” (5) “Acid rain is not related to coal burning;” (6) “Species are interdependent, and the disappearance of one species will produce a chain reaction;” (7) “In the domestic air quality report, tertiary air quality is better than primary air quality;” (8) “Single species of wood are more likely to lead to pest infestations and diseases;” (9) “Class 5 water quality is better than class 1 water quality in the domestic water pollution report;” and (10) “The increase in carbon dioxide in the atmosphere affects climate warming.” A reliability test was conducted, and the Cronbach’s alpha coefficient for the 10 questions was measured as 0.87, showing good internal consistency. The respondents scored 1 point for each correct answer and 0 points for providing incorrect answers or for not knowing the answer, with a cumulative maximum of 10 points and a minimum of 0 points. The higher the score, the stronger an individual’s basic knowledge of the environment, and the lower the score, the more pronounced the individual’s lack of basic knowledge of the environment.

#### Economic level

GDP *per capita* is one of the most important indicators of a region’s macroeconomic development. Regional wealth and economic levels have an important influence on individual green consumption behavior. Green consumption involves a higher level of consumption, and the realization of green consumption requires a certain economic level as a basis. This paper adopts the GDP *per capita* of each province, autonomous region, and municipality directly under the central government in 2010 as a measure of macroeconomic development.

#### Environmental quality

The development of green consumption requires a high-quality ecological environment as an external condition. In the process of urbanization, the greening coverage of built-up areas is an important indicator that reflects the living environment. The greening coverage rate of the built-up area refers to the percentage of the greening coverage area of the built-up area of a city. The greening coverage area refers to the vertical projection area of all vegetation in a city including trees, shrubs, and lawns. The greening coverage rate of built-up areas can reflect the overall environmental construction level of cities, and the environment in which residents live has an important influence on their green consumption behavior (Wolch et al., 2014; Renterghem and Botteldooren, 2016; Song et al., 2020). In this paper, the greening coverage rate of the built-up areas of provinces, autonomous regions, and municipalities directly under the central government in 2010 is used as a measure of overall environmental quality.

#### Other control variables

Other control variables include individual economic ability, social status, education level, political identity, and other basic individual characteristics, which are expressed accordingly by variables such as the logarithm of annual income, education level, political identity, gender, age, and type of household registration.

#### Reliability analysis

We conducted Bartlett’s test of sphericity and calculated the Kaiser–Meyer–Olkin (KMO) measure of sampling adequacy for reliability testing. The results are shown in Table 2. Each variable passed the statistical test of validity.

**Table 2:** Reliability test results of the variables.

Variable	Bartlett test			KMO
	Chi-square	Freedom	value of *p*
Attitudes toward green consumption	10417.428	45	0.000	0.912
Green consumption subjective norms	1677.579	3	0.000	0.659
Green consumption perceived behavioral control	3050.627	15	0.000	0.805
Environmental cognition	12209.753	45	0.000	0.873
Intentions to engage in green consumption	3512.407	15	0.000	0.732

### Method

In society, there is a natural interaction between individuals and the environment. Individuals are influenced by the groups to which they belong and the social environment, and the attributes of the group atmosphere or social environment are also influenced by individuals, who are their constituent elements. The interaction between individuals and the social environment determines the multilayered structure of the data used for this study. The CGSS2010 covers 31 provinces, autonomous regions, and directly administered cities, and because of China’s broad territory and major differences in levels of economic and social development across China’s regions, the heterogeneity of green consumption behavior among individuals in the same region may be significantly lower than that among individuals in different regions, contrary to the classical assumption of the mutual independence and homoskedasticity of error terms used in ordinary least squares (OLS) estimation. Due to this nested data structure resulting from the multistage sampling design of the CGSS2010 and, moreover, to explore the influence of economic and social conditions in different provinces on individual green consumption behavior, this paper adopts a multilayer linear model to empirically test the hypothesis. Specifically, the total variance in the multilayer structure data on the dependent variable is divided into two levels, individual and provincial, and the explanatory variables are introduced separately, where some or all of the estimated parameters of the individual equation are explained as the dependent variables of the province group equation. The relationship between the independent and dependent variables is assumed to be consistent within each province, so the random intercept model is chosen. In this study, a multilevel model including both the individual and province levels of analysis is used to investigate the mechanisms by which environmental cognition influences consumers’ green consumption behavior. To make the coefficients of the intercept practically meaningful at the time of interpretation, the continuous variables of stratum 1 (individual level) and stratum 2 (provincial level) are subjected to total mean alignment in this study. A null model without explanatory variables is estimated to decompose sources of variation in intentions to engage in green consumption behavior into variance between individuals and provinces. The relevant main explanatory variables are included for estimation based on the examination of their intraprovincial correlation coefficients, which are not significantly equal to zero.

Based on the existing research on green consumption from the TPB, we further introduce the variable of environmental cognition to verify the influence of environmental cognition on attitudes toward green consumption, green consumption subjective norms and green consumption perceived behavioral control, as well as its influence on intentions to engage in green consumption behavior and actual green consumption behavior, to clarify the corresponding influence mechanism and provide empirical evidence to help improve policy recommendations to enhance green consumption.

## Results

### Baseline model results

Based on the TPB of green consumption behavior, the mechanism of environmental cognition’s influence on attitudes toward green consumption, green consumption subjective norms, and green consumption perceived behavioral control is further verified, the results of which are shown in Table 3.

**Table 3:** Results of the multilayer linear model of the impact mechanism of green consumption based on the TPB (I).

Dependent variable	(1)	(2)	(3)	(4)	(5)	(6)	(7)	(8)	(9)
	Attitudes toward green consumption			Green consumption subjective norms			Green consumption perceived behavioral control		
**Fixed effect**									
Intercept	0.199 (0.340)	−0.363 (0.582)	−0.303 (0.604)	−0.083 (0.064)	−0.046^***^ (0.208)	0.015^***^ (0.204)	−0.013 (0.195)	−1.092^***^ (0.391)	−1.106^***^ (0.404)
**Individual level**									
Environmental cognition		0.784^***^ (0.041)	0.781^***^ (0.041)		0.064^***^ (0.017)	0.061^***^ (0.017)		0.235^***^ (0.029)	0.234^***^ (0.030)
Gender		0.331^***^ (0.125)	0.332^***^ (0.126)		−0.159^**^ (0.077)	−0.157^**^ (0.077)		−0.023 (0.135)	−0.024 (0.135)
Age		−0.012^*^ (0.006)	−0.013^**^ (0.006)		−0.003 (0.002)	−0.004 (0.002)		−0.008^*^ (0.004)	−0.008^*^ (0.004)
Education level		0.440^***^ (0.105)	0.432^***^ (0.105)		0.081^**^ (0.041)	0.073^*^ (0.041)		0.462^***^ (0.080)	0.463^***^ (0.081)
Logarithm of annual income		0.029 (0.032)	0.028 (0.032)		0.033^***^ (0.011)	0.032^***^ (0.011)		0.046^*^ (0.026)	0.046^*^ (0.026)
Political identity		0.375 (0.342)	0.387 (0.340)		−0.016 (0.113)	−0.002 (0.114)		0.942^***^ (0.220)	0.941^***^ (0.219)
Household registration type		−1.061^***^ (0.210)	−1.037^***^ (0.206)		−0.069 (0.089)	−0.037 (0.088)		−0.208 (0.200)	−0.209 (0.199)
**Provincial level**									
Logarithm of *per capita* GDP			0.481^*^ (0.259)			0.282^***^ (0.094)			−0.019 (0.374)
Greening coverage of built-up areas			−0.033 (0.036)			0.004 (0.010)			−0.019 (0.041)
**Random effect**									
Province	3.045	0.767	0.711	0.083	0.035	0.011	0.952	0.780	0.780
Individual	27.659	20.965	20.966	3.217	3.147	3.148	12.934	11.489	11.488
Intragroup correlation coefficient	9.9%	3.5%	2.3%	2.5%	1.1%	0.3%	6.9%	6.4%	6.4%
*N*	3,194	3,194	3,194	3,194	3,194	3,194	3,194	3,194	3,194

Provincial clustering robust standard errors are in parentheses; ^***^, ^**^, and ^*^ represent significance at the 1%, 5%, and 10% levels, respectively.

Model (1) in Table 3 shows that for the zero model without independent variables for regression, only the intercept term attitude varies randomly across provinces, and the overall linear regression results are not significant. Model (2) adds individual-level variables, and the main explanatory variable, environmental cognition, significantly and positively affects attitudes toward green consumption. The results show that the more comprehensive an individual’s cognition of the environment, the more positive his or her attitudes toward green consumption become, and for every 1-point increase in environmental cognition, attitudes toward green consumption increase by 0.784. The intragroup correlation coefficient is reduced from 9.9% to 3.5% in model (2) relative to model (1). Model (3) further adds the provincial-level explanatory variables of log GDP *per capita* and the green coverage of built-up areas, and the intragroup correlation coefficient further decreases to 2.3%. The explanatory variable at the provincial level, the green coverage of built-up areas, is not significant, indicating that environmental quality has a limited impact on individual attitudes toward green consumption. Log GDP *per capita* is significant, indicating that economic level has a positive and significant impact on individual attitudes toward green consumption. The coefficient signs and significance of other explanatory variables remain unchanged.

Model (4) in Table 3 shows that for the zero model without independent variables for the regression, only the intercept term subjective criterion varies randomly across provinces, and the overall linear regression results are not significant. Model (5) adds individual-level variables, and the intercept term is significant. The main explanatory variable, environmental cognition, significantly and positively affects green consumption subjective norms, and the more comprehensive an individual’s environmental cognition, the stronger green consumption subjective norms become and the greater the pressure on green consumption felt in the outside world. For every 1-point increase in environmental cognition, the green consumption subjective norm value increases by 0.064 points. In model (6), relative to model (5), the explanatory variables of log GDP *per capita* and the green coverage of built-up areas at the provincial level are further added, and the intragroup correlation coefficient is reduced from 1.1% to 0.3%. The log of GDP *per capita*, the explanatory variable at the provincial level, positively and significantly affects green consumption subjective norms, and for each unit increase in the log of GDP *per capita*, green consumption subjective norms increase by 0.282 units. This finding indicates that with an increase in socioeconomic level, the green consumption subjective norms of residents increase, and the external pressure felt by people to engage in green consumption increases, indicating that people residing in more economically developed areas have greater demands for green consumption, while overall environmental quality does not pressure people to engage in green consumption. The significance and sign of the coefficients of the other explanatory variables of green consumption remain unchanged.

Model (7) in Table 3 shows that for the zero model without independent variables for regression, only the intercept term of green consumption perceived behavioral control varies randomly across provinces, and the overall linear regression results are not significant. Model (8) adds variables at the individual level, and the main explanatory variable, environmental cognition, significantly and positively affects green consumption perceived behavioral control, with each 1-point increase in environmental cognition increasing green consumption perceived behavioral control by 0.235 units. This result indicates that the more comprehensive an individual’s cognition of the environment, the easier it becomes for him or her to perceive that he or she has control over the occurrence of green consumption behavior and the less difficult he or she will feel it is to engage in green consumption. In model (8), the intragroup correlation coefficient decreases from 6.9 to 6.4% relative to that in model (7). The explanatory variables at the provincial level, log GDP *per capita* and the green coverage of built-up areas, are further added to model (9). Both explanatory variables at the provincial level are not significant, indicating that economic level (logGDP *per capita*) and environmental quality (green coverage of built-up areas) have limited effects on perceived green consumption by individuals. The significance and coefficient signs of the other explanatory variables remain unchanged.

It is evident that environmental cognition has a significant effect on green consumption when controlling for individual-level variables such as gender, age, and education level and provincial-level variables such as economic level and environmental quality. The deeper the environmental cognition, the more positive the attitude toward green consumption, the stronger the pressure of external norms of green consumption perceived by consumers, and the less difficult the perceived green consumption behavior. According to the TPB, green consumption attitudes, subjective norms, and perceived behavioral control strengthen the formation of intentions to engage in green consumption behavior and thus promote the occurrence of such behavior.

In addition, based on the TPB, it is necessary to further verify the direct influence mechanism of environmental cognition on the intention to engage in green consumption behavior and actual green consumption behavior. This paper also constructs a multilayer linear model for analysis, the results of which are shown in Table 4.

**Table 4:** Results of the multilayer linear model of the impact mechanism of green consumption based on the TPB (II).

	(1)	(2)	(3)	(4)	(5)	(6)
Dependent variable	Intentions to engage in green consumption behavior			Green consumption behavior		
**Fixed effect**						
Intercept	2.177^***^ (0.049)	1.925^***^ (0.104)	1.950^***^ (0.109)	2.230^***^ (0.057)	1.857^***^ (0.214)	1.860^***^ (0.215)
**Individual level**						
Environmental cognition		0.059^***^ (0.006)	0.058^***^ (0.006)		0.040^***^ (0.012)	0.039^***^ (0.013)
Gender		−0.042 (0.028)	−0.041 (0.027)		0.002 (0.046)	0.002 (0.046)
Age		0.003^***^ (0.001)	0.003^***^ (0.001)		0.001 (0.002)	0.001 (0.002)
Education level		0.061^***^ (0.022)	0.058^***^ (0.022)		0.101^***^ (0.039)	0.100^***^ (0.039)
Logarithm of annual income		−0.006 (0.006)	−0.006 (0.006)		0.0004 (0.010)	0.0007 (0.010)
Political identity		0.015 (0.042)	0.019 (0.042)		0.085 (0.072)	0.086 (0.073)
Household register type		−0.147^***^ (0.044)	−0.141^***^ (0.044)		−0.003 (0.070)	−0.0003 (0.070)
**Provincial level**						
Logarithm of *per capita* GDP			0.244^***^ (0.054)			0.075 (0.093)
Greening coverage of built-up areas			−0.013^***^ (0.006)			−0.014 (0.012)
**Random effect**						
Province	0.066	0.030	0.017	0.079	0.068	0.068
Individual	0.450	0.406	0.406	1.350	1.315	1.314
Intragroup correlation coefficient	12.8%	6.9%	4.0%	5.5%	4.9%	4.9%
*N*	3,194	3,194	3,194	3,194	3,194	3,194

Provincial clustering robust standard errors are in parentheses; ^***^, ^**^, and ^*^ represent significance at the 1%, 5%, and 10% levels, respectively.

Model (1) in Table 4 shows that for the zero model without independent variables for regression, intercept term intentions to engage in green consumption vary randomly across provinces, and the overall linear regression results are significant. Model (2) adds individual-level variables, and the main explanatory variable, environmental cognition, positively and significantly affects intentions to engage in green consumption behavior. A 1-unit increase in environmental cognition increases the intention to engage in green consumption by 0.059 units, indicating that the more comprehensive an individual’s cognition of the environment, the more positive his or her intention to engage in green consumption behavior. In model (2), the intragroup correlation coefficient decreases from 12.8 to 6.9% compared to model (1). Model (3) further adds the explanatory variables of the log of GDP *per capita* and green coverage of built-up areas at the provincial level, and the intragroup correlation coefficient further decreases to 4.0%. The two explanatory variables at the provincial level are significant, among which the log of GDP *per capita* is positively significant for an individual’s intentions to engage in green consumption behavior, the logarithm of *per capita* GDP increases by one unit, and green consumption behavior increases by 0.244 units. This finding indicates that the stronger the regional economy, the more likely people will be to have positive green consumption intentions. The green coverage of built-up areas is negatively significant for individual intentions to engage in green consumption behavior, and for each percentage point increase in the green coverage of built-up areas, individual green consumption behavior intention decreases by 0.013, indicating that the stronger the regional environmental quality, the weaker people’s intentions to engage in green consumption. A comfortable environment may offer people less motivation to actively engage in green consumption. The significance and coefficients of the other explanatory variables in model (3) are consistent.

Model (4) in Table 4 shows that for the zero model without independent variables for regression, only the intercept term green consumption behavior varies randomly across provinces, and the overall linear regression results are significant. Model (5) adds individual-level variables, and the intercept term is significant. The main explanatory variable, environmental cognition, significantly and positively affects green consumption behavior, and with a 1-unit increase in environmental cognition, green consumption behavior increases by 0.040. The more comprehensive an individual’s cognition of the environment, the more likely he or she will be to engage in green consumption behavior. Among the other individual-level control variables, the higher the education level, the higher the likelihood of green consumption behavior, and for each increase in the level of education, the green consumption behavior of residents increases by 0.101. The correlation coefficient within the group is reduced from 5.5% to 4.9% in model (5) compared to model (4). The explanatory variables of the log of GDP *per capita* and green coverage of built-up areas at the provincial level are again added to model (6). The explanatory variables of the green coverage of built-up areas and GDP *per capita* at the province level do not have significant effects on green consumption behavior, indicating that regional environmental quality and economic level do not directly determine whether individual green consumption behavior exists. The coefficients of the other variables are consistent and significant with model (5).

First, we can see that the deepening of individual environmental cognition can not only promote the intention to engage in green consumption but also directly push individuals to engage in green consumption behavior. Given that environmental cognition has a positive and significant effect on green consumption attitudes, the deepening of environmental cognition can reduce the attitude-behavior inconsistencies of an individual’s green consumption to a certain extent and more often transform green consumption attitudes into green consumption behavioral practice. However, as shown above, education level and household type have significant effects on individuals’ attitudes toward green consumption, and the higher the education level, the more positive individuals’ attitudes toward green consumption. Individuals in nonagricultural households have more positive attitudes toward green consumption than those in agricultural households.

Second, the green coverage of built-up areas, which represents environmental quality, can inhibit an increase in residents’ intentions to engage in green consumption. This result means that an increase in environmental quality prompts people to reduce their cognition regarding green consumption, attributing the positive externality effect of green consumption to the consumption behavior of others.

### Endogeneity discussion

On the one hand, environmental cognition positively influences attitudes toward green consumption, green consumption subjective norms, green consumption perceived behavioral control, intentions to engage in green consumption behavior, and actual green consumption behaviors. On the other hand, having a stronger attitude toward green consumption, green consumption subjective norms, green consumption perceived behavioral control, intentions to engage in green consumption behavior, and actual green consumption behaviors may often lead to stronger environmental cognition. Considering the possible two-way causality and omission of variables in this study and the resulting impact of endogeneity problems on the estimation results, a two-stage least squares (2SLS) regression is performed on the original model by introducing IVs to avoid endogeneity problems as much as possible.

First, this paper selects terrain undulation, precipitation, and the percentage of higher education graduates in each province as IVs of environmental cognition to verify the effect of environmental cognition on attitudes toward green consumption. China is a vast country with diverse topography, and terrain undulation is an important factor influencing the distribution of population and labor intensity in China, while areas of low topographic relief (e.g., plains) are mostly populated areas with faster economic, social, and cultural development and stronger environmental cognition. In remote mountainous areas and plateaus of high topographic relief, the development of economic, social, and cultural aspects lags behind, and people have less environmental knowledge. Similarly, areas with more rainfall in China tend to be more livable; have developed inland navigation; and exhibit faster economic, social, and cultural development; and residents of these areas may have a better understanding of the environment. Therefore, topographic relief and precipitation by province serve as important indicators with which to portray the natural environment of a region and as IVs for environmental cognition. The number of higher education graduates in each province reflects the level of education in a region, which affects an individual’s perception of the environment. At the same time, terrain undulation, precipitation, and the percentage of higher education graduates in each province do not directly influence individuals’ attitudes toward green consumption but are objective indicators that satisfy the exogeneity of the IVs.

Second, this paper uses the groundwater potential of each province as an IV of environmental cognition to verify the influence of environmental cognition on subjective guidelines of green consumption. The groundwater potential^1^ of each province is an important indicator with which to portray the natural environment, and an abundance of groundwater may influence people’s perceptions of the environment by affecting their daily production and living environment. At the same time, the groundwater potential in each province is an objective indicator, and green consumption subjective norms do not have a direct correlation with groundwater potential, satisfying the condition of IV exogeneity.

Third, precipitation by province and the overall “environment” index from Baidu are selected as the IVs of environmental cognition to verify its influence on the perceived behavioral control of green consumption. Similarly, precipitation in each province and the overall Baidu index for the “environment” are objective natural environmental and environmental cognition-related indicators related to people’s environmental cognition but that do not directly affect people’s green consumption. Therefore, they satisfy the condition of IV exogeneity.

Fourth, this paper selects groundwater potential, precipitation, and Baidu’s overall index^2^ for the “environment” for each province as the IVs of environmental cognition to verify the influence of environmental cognition on intentions to engage in green consumption behavior. Groundwater potential, precipitation, and the overall Baidu index regarding the “environment” for each province can directly reflect local populations’ cognition of the environment and are correlated; however, these indicators do not directly affect people’s intentions to engage in green consumption behavior, satisfying the condition of IV exogeneity.

Fifth, groundwater potential, precipitation, and Baidu’s overall “environment” index for each province are selected as the IVs of environmental cognition to verify the influence of environmental cognition on green consumption behaviors. Similarly, groundwater potential, precipitation, and Baidu’s overall “environment” index for each province satisfy both the correlation condition with environmental cognition and the exogeneity condition with green consumption behavior.

In addition, when IVs are introduced to perform 2SLS regression in the original model, each IV passes the overidentification test and weak IV test in the regression, the results of which are shown in Table 5.

**Table 5:** IV-2SLS estimation results.

	(1)	(2)	(3)	(4)	(5)
Dependent variable	Attitudes toward green consumption	Green consumption subjective norms	Green consumption perceived behavioral control	Intentions to engage in consumption behavior	Green consumption behaviors
Environmental cognition	1.780^***^ (0.673)	0.295^*^ (0.158)	0.026^***^ (0.009)	0.196^***^ (0.017)	0.154^**^ (0.075)
Other individual and provincial variables	YES	YES	YES	YES	YES
*N*	3,240	3,240	3,240	3,240	3,240
**First-stage regression**					
Terrain undulation (IV)	−0.070 (0.11)		−0.032 (0.051)		
Groundwater potential (IV)		−0.004^***^ (0.001)		−0.004^**^ (0.002)	−0.004^***^ (0.001)
Precipitation (IV)	−0.001^***^ (0.0003)		−0.0005^***^ (0.0001)	−0.0005^*^ (0.0003)	−0.0005^***^ (0.0001)
Percentage of higher education graduates (IV)	−2.519^***^ (0.864)				
Baidu’s overall index for the “environment” (IV)		0.005 (0.008)	0.011^***^ (0.004)	0.010 (0.009)	0.010^***^ (0.004)
First-stage regression F-value	79.85	77.46	111.92	68.36	122.08

Provincial clustering robust standard errors are in parentheses; ^***^, ^**^, and ^*^ represent significance at the 1%, 5%, and 10% levels, respectively.

**Figure 2:**
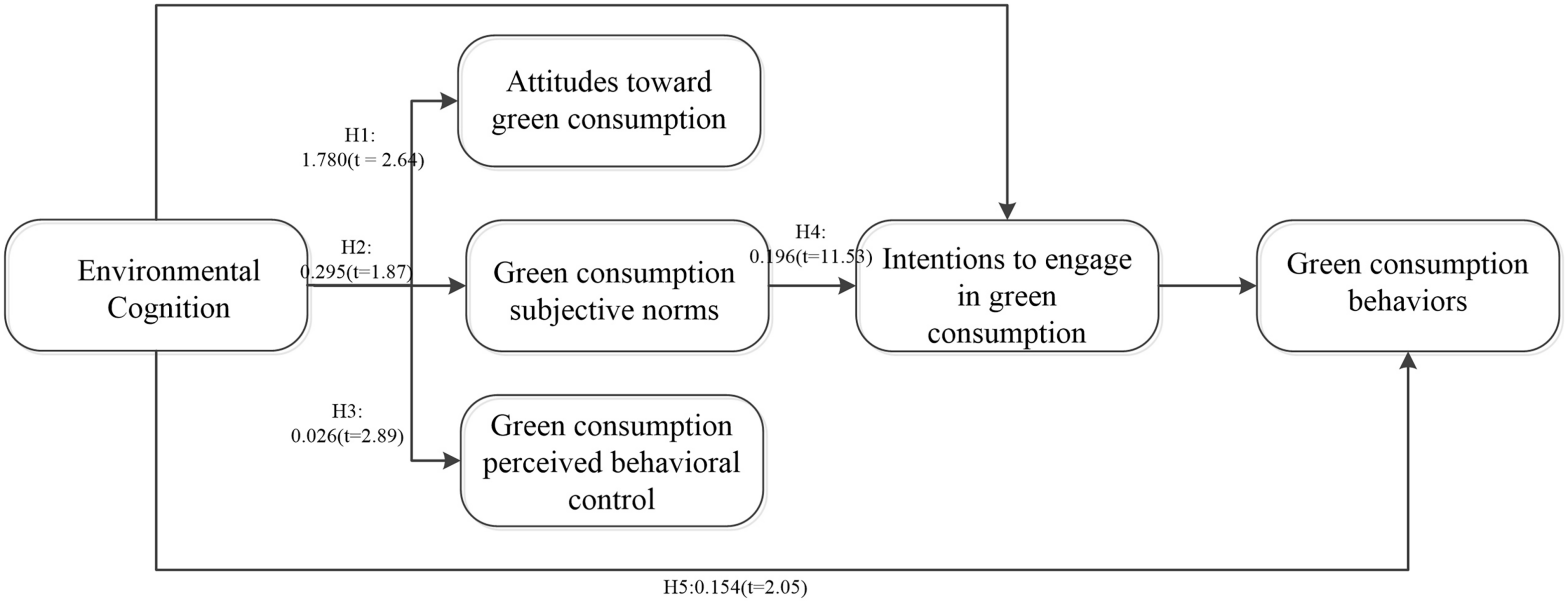
TPB model of green consumption behavior with values.

The results in Table 5 show that environmental cognition has a significant positive impact on variables such as attitudes toward green consumption, green consumption subjective norms, and green consumption perceived behavioral control. The more comprehensive people’s cognition of the environment, the more positive their attitudes toward green consumption; with a 1-unit increase in environmental cognition, attitudes toward green consumption increase by 1.78 units. The more comprehensive people’s cognition of the environment, the greater the external pressure they feel regarding green consumption; with a 1-unit increase in environmental cognition, green consumption subjective norms increase by 0.295 units. The more comprehensive people’s cognition of the environment, the less difficult it will be for them to engage in green consumption behaviors; with a 1-unit increase in environmental cognition, green consumption perceived behavioral control increases by 0.026 units. The above findings are consistent with the baseline regression model. According to the empirical results, we demonstrate these hypotheses and show in Figure 2.

### Robustness check

To determine whether individuals’ environmental cognition influences green consumption according to the TPB, this paper conducts several robustness tests on the mechanism path.

First, we consider the considerable differences in China’s regional economic and social environment. For example, the central and eastern regions enjoy certain preferential regional development policies. To test the possible influence of the confounding factors brought about by important differences in policies, dummy variables such as eastern and central regions are added, the specific results of which are shown in Tables 6, 7.

**Table 6:** Estimation results of the multilayer linear model considering the eastern region.

	(1)	(2)	(3)	(4)	(5)
Dependent variable	Attitudes toward green consumption	Green consumption subjective norms	Green consumption perceived behavioral control	Intentions to engage in consumption behavior	Green consumption behaviors
Environmental cognition	0.783^***^ (0.04)	0.061^***^ (0.017)	0.224^***^ (0.03)	0.059^***^ (0.005)	0.039^***^ (0.013)
Control variable	YES	YES	YES	YES	YES
Intercept	−0.92 (0.545)	0.001 (0.191)	−1.143^*^ (0.588)	1.875^***^ (0.114)	1.821^***^ (0.235)
*N*	3,240	3,240	3,240	3,240	3,240

Provincial clustering robust standard errors are in parentheses; ^***^, ^**^, and ^*^ represent significance at the 1%, 5%, and 10% levels, respectively.

**Table 7:** Estimation results of the multilayer linear model considering the central region.

	(1)	(2)	(3)	(4)	(5)
Dependent variable	Attitudes toward green consumption	Green consumption subjective norms	Green consumption perceived behavioral control	Intentions to engage in consumption behavior	Green consumption behaviors
Environmental cognition	0.780^***^ (0.041)	0.061^***^ (0.017)	0.234^***^ (0.03)	0.058^***^ (0.006)	0.039^***^ (0.012)
Control variable	YES	YES	YES	YES	YES
Intercept	−0.166 (0.637)	0.012 (0.203)	−1.047^***^ (0.431)	1.966^***^ (0.11)	1.899^***^ (0.215)
*N*	3,240	3,240	3,240	3,240	3,240

Provincial clustering robust standard errors are in parentheses; ^***^, ^**^, and ^*^ represent significance at the 1%, 5%, and 10% levels, respectively.

The results in Tables 6, 7 show that environmental cognition produces significant positive effects on attitudes toward green consumption, green consumption subjective norms, and green consumption perceived behavioral control, which then promote green consumption behavior in both the central and eastern regions. At the same time, environmental cognition also has a significant positive effect on intentions to engage in green consumption behavior and green consumption behavior. This series of results suggests that deeper and more comprehensive environmental cognition is still an important factor in encouraging individuals to engage in green consumption behaviors under preferential ecological and environmental policies adopted in the central and eastern regions.

Second, to verify whether environmental cognition has a regional differential effect on green consumption behavior, an interaction term between environmental cognition and regions is included, the estimated results of which are shown in Table 8.

**Table 8:** Estimation results of the multilayer linear model considering regional heterogeneity.

	(1)	(2)	(3)	(4)	(5)
Dependent variable	Attitudes toward green consumption	Green consumption subjective norms	Green consumption perceived behavioral control	Intentions to engage in consumption behavior	Green consumption behaviors
Environmental cognition	0.114^***^ (0.027)	0.114^***^ (0.027)	0.214^***^ (0.046)	0.051^***^ (0.01)	0.055^***^ (0.011)
Environmental cognition*northeastern region	−0.046 (0.161)	−0.046 (0.033)	0.029 (0.068)	−0.027^*^ (0.016)	−0.044 (0.051)
Environmental cognition*Central region	−0.105^***^ (0.034)	−0.105^**^ (0.034)	−0.012 (0.085)	0.019 (0.014)	−0.0005 (0.051)
Environmental cognition* western region	−0.079^**^ (0.033)	−0.079^**^ (0.033)	0.076 (0.067)	0.024^*^ (0.013)	−0.034 (0.023)
Control variable	YES	YES	YES	YES	YES
Intercept	0.131 (0.254)	0.121 (0.254)	−1.334^**^ (0.571)	2.032^***^ (0.124)	1.922^***^ (0.161)
*N*	3,240	3,240	3,240	3,240	3,240

Provincial clustering robust standard errors are in parentheses; ^***^, ^**^, and ^*^ represent significance at the 1%, 5%, and 10% levels, respectively.

The results in Table 8 show that environmental cognition has a significant positive effect on attitudes toward green consumption, green consumption subjective norms, green consumption perceived behavioral control, intentions to engage in green consumption behavior, and green consumption behaviors when regional differences in the influencing factors of green consumption are considered. When examining the influence of environmental cognition on attitudes toward green consumption and subjective norms, this paper finds that this influence is significantly stronger in the eastern region than in the central and western regions, indicating that the increase in the level of environmental cognition in the eastern region is more conducive to the formation of attitudes toward green consumption than in the central and western regions. People in the eastern region feel more external pressure to adopt green consumption, indicating that in addition to improving environmental cognition, it is also necessary to pay corresponding attention to the central and western regions. This result suggests that in the central and western regions, in addition to raising the level of environmental cognition, attention should be given to improving the overall cultural quality of the region, refining the green consumption rules and regulations, and strengthening the implementation of the reward and punishment system. In terms of the influence of environmental cognition on intentions to engage in green consumption, the eastern region is significantly stronger than the northeastern region and weaker than the western region, indicating that the direct promotion of intentions to engage in green consumption by improving environmental cognition is most effective in the western region and limited in the northeastern region. The results of this paper have more specific policy implications for improving regionally differentiated green consumption guidance policies.

## Discussion

Based on the TPB framework, this study uses multilayer linear analysis and household microdata to test the influence of environmental cognition on consumers’ green consumption behaviors and the path mechanisms and draws the following conclusions.

First, in terms of individual characteristics and behavioral intentions at the individual level, environmental cognition, as an antecedent variable, can positively influence three variables—attitudes toward green consumption, green consumption subjective norms, and green consumption perceived behavioral control—which, in turn, lead to intentions to engage in green consumption behavior and actual green consumption behaviors. Environmental cognition can also lead to green consumption behaviors by promoting intentions to engage in green consumption behavior or directly promoting green consumption behavior.

Second, from the perspective of economic levels and environmental quality at the provincial level, an elevated regional level of economic development positively influences attitudes toward green consumption, green consumption subjective norms, and intentions to engage in green consumption behavior. The more developed the regional economic level, the stronger people’s attitudes toward green consumption, and the more external constraints related to environmental protection, such as green consumption, are imposed; thus, people in these regions may perceive more external pressure to take up green consumption. The higher the economic level of a region, the more likely people will be to have intentions to engage in green consumption behavior; however, this level does not have a direct impact on green consumption behavior. In contrast, the environmental quality of the region somewhat inhibits the increase in intentions to engage in green consumption behavior, and people consider the positive externality effects of green consumption on the consumption behavior of others.

Third, the influence of environmental cognition on green consumption shows regional heterogeneity. The influence of environmental cognition on attitudes toward green consumption and subjective norms is significantly stronger in the eastern region than in the central and western regions, indicating that people’s environmental cognition level in the eastern region is more conducive to the formation of attitudes toward green consumption than in the central and western regions and that people in the eastern region feel more pressure from society to take part in green consumption. The influence of environmental cognition on intentions to engage in green consumption is significantly greater in the eastern region than in the northeastern region and weaker than in the western region.

## Theoretical implications

First, this study enriches the path of the green consumption behavior mechanism based on the TPB. Previous research has proven that the TPB is more effective in studying green consumption behavior. This paper takes environmental cognition as a predetermined variable to test how it affects green consumption behavior. This study explains another potential reason for the inconsistent impact of attitude on behavior in the micro mechanism of green consumption behavior, and it is also another expansion and application of the TPB in research on green consumption behavior.

Second, different from previous studies, which focused on analyzing the impact of factors such as consumers’ environmental attitudes and payment ability as well as product eco-labels on green consumption behavior from a micro perspective, this paper also supplements the impact mechanism of green consumption behavior from a macro perspective. This study finds that provincial economic and environmental factors have different effects on consumers’ green consumption intentions and behavior. It is not simply the case that a high level of regional economic development or good environmental quality leads to more green consumption behaviors.

Third, in the past, few studies were carried out to analyze the regional heterogeneity of the impact of China’s environmental cognition on green consumption. However, the economic development level, environmental quality, the overall cultural quality of residents, and the environmental protection laws and regulations are different in different regions. Therefore, there are differences in the effectiveness of the mechanism of impact on green consumption, and it is necessary to formulate differentiated green consumption development policies and measures for different regions.

## Managerial implications

Based on the results of the study and taking into account the actual state of green consumption development in China and the requirements of sustainable economic development, the following recommendations are made for the future development of green consumption in China.

First, an accurate concept of environmental protection should be established. In the process of transforming nature, the value of reverence for nature should be seen as important. We must realize that natural resources are not inexhaustible and that the overexploitation of natural resources will inevitably lead to a shortage of resources needed for basic survival and development in the future. We should formulate scientific and reasonable policies for natural resource protection to ensure the renewable nature of natural resources and achieve intergenerational equity. It is necessary to reverse the idea of excessive consumption through social opinion and administrative means; resist trends of hedonism; apply high taxes to wasteful phenomena; and promote the value of green, environmentally friendly, and economical consumption.

Second, it is necessary to optimize the design process of relevant policies. For policymakers, developing a standardized process to a certain extent can ensure that policies are as environmentally friendly as possible. To that end, there are two aspects. The first is to introduce a compulsory discussion about the effects of policies on the environment into the policy-making process. The second is to set certain rewards and punishment regulations so that people who engage in green consumption behavior in life can enjoy economic or other compensation and people who commit violations in regard to green consumption behavior can pay costs.

Third, differentiated green consumption development strategies should be developed by taking advantage of the higher levels of environmental awareness and economic development in the eastern region, accelerating research on carbon derivative product innovation in conjunction with the financial sector, and launching carbon futures and other derivative products in a timely manner to stabilize investors’ price expectations and reduce carbon trading market risks. In view of the shortcomings of environmental literacy, the policy environment, and economic levels in the central and western regions, we will promote green consumption education from the government to enterprises, from students to adults, from consumers to producers, and from towns to villages. Moreover, special funds will be used to guide green consumption education in remote areas, transform green consumption education from a phased activity to a long-term, systematic project, and establish a three-in-one (school-unit-community) education system pattern.

## Limitations and future research venues

First, this paper uses data from 2010 to empirically analyze the effect of environmental cognition on green consumption behavior. The multilayer linear method and IVs are employed to identify the causal relationship between environmental cognition and green consumption behavior. We tend to believe that the identified causal relationship is a social law rather than a phenomenon; thus, it is stable. However, considering the enormous impact of the COVID-19 pandemic, if we can take post-pandemic data for further verification, doing so will enhance the credibility of the conclusions.

Second, this paper uses cross-sectional data. Thus, it is impossible to control for the unobservable fixed effects of individuals and to track and predict the behavior of individuals. Conducting analysis again when panel data are available in the future is an important direction for improvement.

Third, due to the limitation of data availability, the measurement of green consumption behavior in this paper is relatively simplified. In future research, quantifying the multidimensional connotation of green consumption behavior systematically and in depth is an important direction. Distinguishing between private-sphere and public-sphere green consumption behavior will have important theoretical and managerial implications.

## Data availability statement

Publicly available datasets were analyzed in this study. This data can be found at: http://www.cnsda.org/index.php?r=projects/view&id=15553986

## Author contributions

CX and RW: conceptualization. CX: methodology, formal analysis, writing—original draft preparation, and funding acquisition. RW: software. CX, RW, and XG: validation. XG: data curation. CX and XG: writing—review and editing. All authors contributed to the article and approved the submitted version.

## Funding

This work was supported by the Fundamental Research Funds for the Central Universities (JBK2201057).

## Conflict of interest

The authors declare that this research was conducted in the absence of any commercial or financial relationships that could be construed as a potential conflict of interest.

## Publisher’s note

All claims expressed in this article are solely those of the authors and do not necessarily represent those of their affiliated organizations, or those of the publisher, the editors and the reviewers. Any product that may be evaluated in this article, or claim that may be made by its manufacturer, is not guaranteed or endorsed by the publisher.

## Footnotes

^1^Data source: China Natural Resources Series (Mineral Volume), Beijing: China Environmental Science Press, 1996 edition.

^2^The overall "environment" index from Baidu represents the frequency with which residents search for the word "environment" on Baidu. The larger the index is, the greater the residents’ cognition of the environment. The detailed information is shown on the website: http://index.baidu.com/v2/index.html.
